# The angiotensin-converting enzyme inhibitor, captopril, suppressed hepatic stellate cell activation via NF-kappaB or wnt3α/β-catenin pathway

**DOI:** 10.1080/21655979.2021.1987091

**Published:** 2021-10-20

**Authors:** Zhaodi Gu, Linjun Fang, Peijun Ma

**Affiliations:** Internal Medicine Department, Shaoxing Yuecheng People’s Hospital, Shaoxing City, Zhejiang Province, China

**Keywords:** Captopril, hepatic fibrosis, high glucose, nf-kappaB, wnt3α/β-catenin

## Abstract

Activation of hepatic stellate cells (HSC) is associated with hepatic fibrogenesis, which is one of complications of diabetes mellitus. Captopril possesses potent anti-inflammation, oxidative stress and fibrosis effects. However, the specific molecular mechanism of captopril in high glucose (HG)-induced hepatic stellate cells has not been elucidated. Following the treatment of HG or captopril treatment for rat hepatic stellate cells (HSC-T6), cell activities were detected by Cell Counting Kit-8 (CCK8) assay. Reactive oxygen species (ROS) levels were determined by ROS staining. The expression of inflammation-related proteins (Interleukin (IL)-1β, IL-6 and IL-8) and fibrosis-related proteins (fibronectin (FN), collagen I, collagen III, collagen IV, matrix metallopeptidase (MMP-2 and MMP-9) were determined by Western blot. Captopril significantly decreased HSC-T6 cell viability induced by HG in a dose-dependent manner, as well as decreased levels of malondialdehyde (MDA), ROS, pro-inflammatory markers and fibrosis-related proteins, while upregulated superoxide dismutase (SOD) activities. We further found that captopril decreased the ratio of p-IκBα/IκBα and the ratio of p-p65/p65. Intriguing, phorbol myristate acetate (PMA) or LiCl was able to significantly reverse the captopril-induced alteration of oxidative stress-, inflammation- and fibrosis-marker levels. In conclusion, in HG-stimulated HSC-T6 cells, captopril displayed a potent ability to inhibit oxidative stress, inflammation and hepatic fibrogenesis via NF-kappaB or wnt3α/β-catenin. These results demonstrated the mechanism of captopril as well as the role of the NF-kappaB or wnt3α/β-catenin on HSC-T6 activation induced by HG.

## Introduction

Hepatic fibrosis is one of the many complications of diabetes mellitus. Hepatic stellate cells (HSCs) are the main cell source for the generation of extracellular matrix when liver injury occurs and play a central role in the formation of liver fibrosis [1,2]. The activation process of hepatic stellate cells can be divided into initiation stage and continuous activation stage. If the activation process can be blocked in time, the further development of the disease can be blocked, and the result of hepatic fibrosis can be terminated or even reversed [3–5].

Captopril is an inhibitor of angiotensin converting enzyme (ACE), which is commonly used to low blood pressure in patients with high blood pressure [6]. Captopril combined with gliclazide has been found to reduce vascular and renal complications and control blood glucose in streptozotocin-induced diabetic rats [7]. In addition, captopril has a protective effect on renal injury caused by ischemia reperfusion in diabetic rats [8]. These studies suggest that captopril may work against diabetes. In addition, captopril alleviates renal injury and inflammation in spontaneously hypertensive rats through NF-κB signaling pathway [9], which also is reported to improve fibrosis and oxidative stress of lipopolysaccharide-induced pneumonia [10]. In addition, some studies have reported that captopril plays a protective role in diabetic liver injury [11], and can reduce TAC-induced heart failure by inhibiting Wnt3A/β-catenin and JAK2/STAT3 pathways [12]. It is reported that the activation of Wnt/β-catenin signaling pathway can promote liver fibrosis [13]. However, the specific molecular mechanism of captopril in HG induced hepatic stellate cells has not been studied. Taken together, we assume that NF-κB and Wnt/β-catenin signaling pathways could be involved in the effects of captopril on HG-induced hepatic stellate cells. Therefore, we further explore the mechanism of captopril in HG-induced HSCs, and speculate that captopril can reduce the abnormal proliferation, inflammatory response and fibrogenesis of HG-induced HSCs by inhibiting NF-κB and Wnt/β-catenin signaling pathways.

## Method

Cell culture

HSC-T6 cells were purchased from ATCC (USA) and cultured in DMEM medium (Hyclone, USA) containing 10% FBS (Gibco, USA) in 37 °C with 5% CO2. HSC-T6 was subjected to glucose treatment at a concentration of (0, 10, 30, 50, 70, 100 mM) to observe cell activity, inflammatory cytokines and ROS production. In the next experiment, HSC-T6 cells were pretreated with different concentrations of captopril (0, 0.1, 0.3, 0.5, 1, 3 mM) for 12 h, and incubated with HG (50 mM) for 48 h.

CCK8 assay

HSC-T6 was seeded into 96-well plates with 2 × 10^4^ cells per well. After high glucose or captopril treatment, or pretreatment with different concentrations of captopril for 12 h and then incubated with HG (50 mM) for 48 h, the cell viability was detected using CCK8 kit according to manufacturer’s guidance (ThermoFisher. Inc). 10 µL CCK8 solution was added to each well for incubation for 2 h. The absorbance at 450 nm was detected using a microplate reader (ThermoFisher. Inc).

The detection of SOD activity and MDA levels

HSC-T6 cells were collected and washed with PBS of 4°C. The SOD activities were detected using Total SOD activity detection kit (Beyotime, Shanghai, China). For the detection of MDA, cells were collected and lysed using RIPA lysis buffer (ThermoFisher. Inc). Then, the supernatant was collected after centrifugation at 12000 g for 10 min. The MDA activities were detected using MDA detection kit (Beyotime, Shanghai, China).

ROS staining

The ROS was detected using Reactive oxygen species assay kit (Beyotime, Shanghai, China) according to manufacturer protocol. The cells were collected and suspended in DCFH-DA. After incubation at 37°C for 20 min, the cells were observed under a fluorescence microscope (OLYMPUS).

Quantitative reverse transcription PCR (RT-qPCR) assay

The total RNA of cells was extracted with FAST200 kit. The concentration and purity of RNA were determined by spectrophotometer and cDNA was synthesized according to the Fermentas Reverse Transcription Kit instructions. The cDNA was amplified by SYBR® Premix Ex Taq (TAKARA, Japan). The primers were designed using the gene sequences provided in NCBI Genebank and synthesized by Beijing Aoko Biological Engineering Co., Ltd. The used primers used in this study are as following: IL-6 forward 5ʹ- CCACCAGGAACGAAAGTCAAC-3ʹ, reverse 5ʹ- GGCAGTGGCTGTCAACAACA-3ʹ; IL-1β forward 5ʹ- GACCTGTTCTTTGAGGCTGAC-3ʹ, reverse 5ʹ- TCCATCTTCTTCTTTGGGTATTGTT-3ʹ; IL-8 forward 5ʹ-TGGTCTCAGCCACCCGC-3ʹ, reverse 5ʹ-TCCACGACATACTCAGCA −3ʹ;

β-actin forward 5ʹ- GCTCGTCGTCGACAACGGCTG- 3ʹ, reverse 5ʹ-CAAACATGATCTGGGTCATCTTTTC-3ʹ. β-actin was used to be as an internal reference.

Enzyme Linked Immunosorbent Assay (ELISA)

The cells were seeded into 6-well plate. After the intervention of high glucose, the supernatant of cell culture medium was collected and centrifuged at 3000 r/min for 15 min to collect supernatant. The experiment was performed to detect the levels of IL-1β, IL-6 and IL-8 in strict accordance with the kit instructions (Beyotime Biotechnology, Shanghai, China).

Western blot

Total protein was extracted by protein extraction kit, and then electrophoresis was performed in polyacrylamide gel. The protein was electrically transferred to PVDF membrane, blocked with 5% skimmed milk powder and incubated with primary antibodies (FN, ab268020; collagen I, ab270993; collagen III, ab275881; collagen IV, ab270993; MMP-2, ab92536; MMP-9, ab76003; GAPDH, ab8245. Abcam, England) at 4 °C overnight, followed by the incubation with HRP-conjugated secondary antibodies (ab7090, Abcam, England) for 2 h at 37°C. GAPDH was used as an internal reference. The optical density ratio of target protein to corresponding internal reference was calculated by Image J software 1.46 r.

Statistical analysis

Prism 7.0 software was used for statistical analysis of experimental data. Data were represented by mean ± standard deviation (SD). One-way analysis of variance was used to determine the difference among different groups, followed by hos turkey’s test. p < 0.05 indicates that the difference is statistically significant.

## Result

Captopril suppressed HSC-T6 cell activities induced by HG

**Figure 1.**
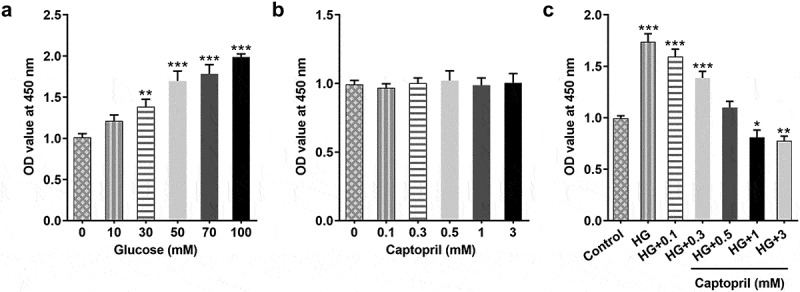
Captopril reduced HSC-T6 cells viability. (a) After HSC-T6 cell was exposed to glucose of different concentration for 48 h, cell viability was detected through CCK8 assay. (b) CCK8 assay. (c) CCK8 assay. *p < 0.05, **p < 0.01, *** p < 0.001

To investigate the effects of captopril on HSC-T6 cell subject to HG, we assessed HSC viability after captopril treatment using CCK8 assay. Different concentrations of HG were used to stimulate HSC. The results showed that with the increase in HG concentration, the cell viability of HSC was significantly increased (Figure 1a). In order to evaluate the cytotoxic effect of captopril on HSC-T6, HSC-T6 cells were treated with different concentrations of captopril. There were no significant effects for captopril on cell viability (Figure 1b). Then, we further found that captopril reduced viability of HSC challenged with HG in concentration-dependent manner (Figure 1c). In subsequent experiments, 1 mM of captopril was used for deeper mechanism exploration for its effects on HSC in response to HG.

Captopril suppressed oxidative stress, inflammation and fibrosis in HSC-T6 cells exposed to HG

**Figure 2.**
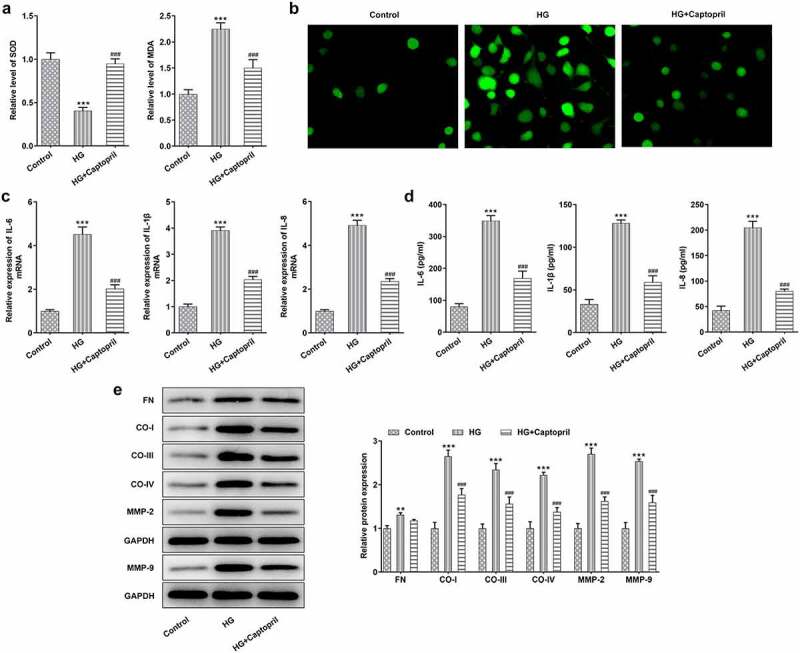
Captopril reduced oxidative stress, inflammation and fibrosis in HG-treated HSC-T6. (a) The evaluation of SOD and MDA levels through related kit. (b) ROS staining results. (c) The levels of liver fibrosis-related proteins were detected through Western blot. FN: fibronectin, CO-I: collagen I, CO-III: collagen III, CO-IV: collagen IV. *** p < 0.001 Vs Control. ^###^p < 0.001 Vs HG

To analyze the mechanism of captopril in HSC-T6 cells challenged with HG, the effects of captopril on oxidative stress, inflammation and fibrosis in HSC-T6 cells cotreated with HG and captopril were determined. The results showed that captopril exhibited markedly higher SOD activities and SOD levels than HG treatment alone, while reverse effects were observed in MDA levels (Figure 2 A). We observed an obvious decrease in fluorescence intensity for ROS staining after captopril treatment (Figure 2b). By testing the expression of inflammation-related factors through qPCR and ELISA assay, the results showed that captopril treatment markedly lessened the levels of IL-1β, IL-6 and IL-8 (Figure 2c-d) as relative to HG group. The levels of FN, collagen-I, collagen-III, collagen-IV, MMP-2 and MMP-9, which have been recognized to be as markers for liver fibrosis, were also analyzed by Western blot assay in HG-challenged HSC-T6 cells following captopril treatment. As shown in Figure 2e, these protein levels were markedly decreased when captopril was used to co-treat cells with HG.

Captopril inhibits HG-induced activation of the NF-kappaB and wnt3a/β-catenin pathway in HSC-T6 cells

**Figure3:**
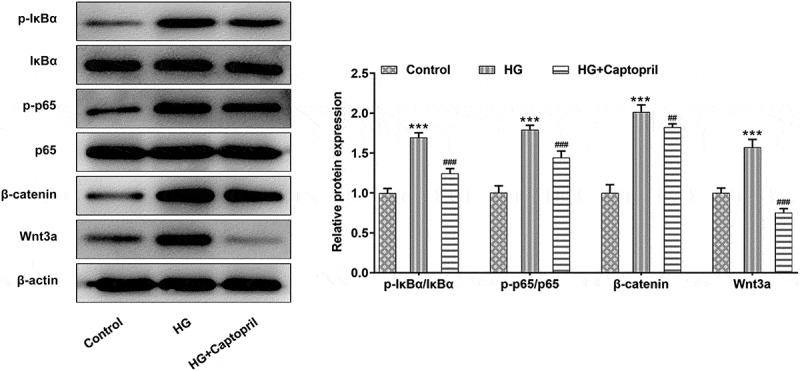
The detection of NF-kβ and wnt3a/β-catenin pathway in HG-induced HSC-T6 cells. *** p < 0.001 Vs Control. ^##^p < 0.01, ^###^p < 0.001 Vs HG

To analyze the role of the NF-kappaB and wnt3a/β-catenin after captopril treatment, we looked at expression levels of NF-kappaB pathway-related proteins and wnt3a/β-catenin. The phosphorylation of IκBα makes it possible that NF-κB is released from the cytoplasmic NF-κB/IκBa complex and activated to expose the nuclear localization domain and p65 is confirmed to be the main transcriptional factor. Increased ratio of p-IκBα/IκBα and the ratio of p-p65/p65 are considered to be related to be involved in NF-κB activation [14–16]. As shown in results, captopril markedly decreased the ratio of p-IκBα/IκBα and the ratio of p-p65/p65 when compared with HG alone (Figure 3). In addition, the expression of Wnt3A and β-catenin were detected. The results showed that the expression of Wnt3a/β-catenin protein was increased after HG treatment, while that of Wnt3a/β-catenin was decreased after captopril treatment (Figure 3). The above results indicated that captopril could inhibit HG-induced activation of NF-kappaB pathway and Wnt3A/β-catenin pathway in HSC-T6 cells.

Captopril inhibited HG-induced oxidative stress, inflammatory response, and fibrosis in HSC-T6 cells by blocking the NF-κB pathway and Wnt3α/β-catenin pathway

**Figure 4.**
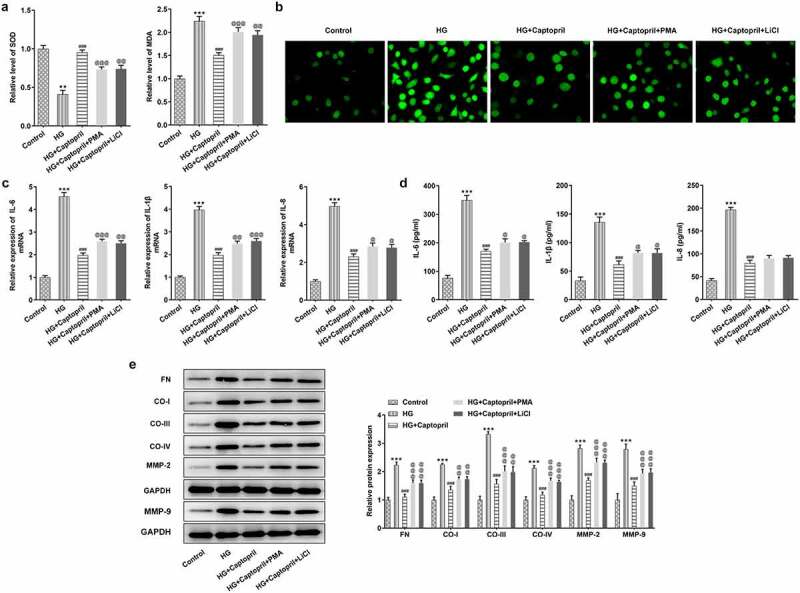
PMA or LiCl treatment reversed the effects of captopril in oxidative stress, inflammatory response, and fibrosis. (a) The detection of SOD and MDA levels by related kits. (b) SOD staining. (c) The analysis of inflammatory factors through qPCR. (d) The detection of inflammatory factors levels by Western blotting. (e) The evaluation of liver fibrosis-related markers by Western blot assay. FN: fibronectin, CO-I: collagen I, CO-III: collagen III, CO-IV: collagen IV. **p < 0.01, *** p < 0.001 Vs Control. ^@^ p < 0.05, ^@@^ p < 0.01, ^@@@^ p < 0.001 Vs HG+Captopril

Next, whether the effects of captopril on oxidative stress, inflammatory response, and fibrosis in HSC-T6 cells were related to NF-κB pathway and Wnt3α/β-catenin pathway was further investigated. We used NF-κB activator or Wnt signaling pathway activator to determine whether captopril affected NF-κB signaling pathway and Wnt signaling pathway to suppress oxidative stress, inflammation and fibrosis. Accumulating evidences have confirmed that PMA is able to activate NF-κB pathway and LiCl is an activator of Wnt signaling pathway [17–22]. Treatment with PMA (NF-κB activator) or treatment with LiCl Wnt signaling pathway activator) resulted in a significant decrease in SOD activities and an increase in MDA levels compared with the cotreatment group of HG and captopril (Figure 4a). DCFH-DA staining for ROS demonstrated marked attenuation in green fluorescence intensity after PMA or LiCl treatment (Figure 4b). Next, we examined the effects of PMA or LiCl on inflammatory markers. PMA or LiCl treatment significantly partly recovered captopril-treated effects on levels of IL-1β, IL-6 and IL-8 in HG-induced HSC-T6 cells (Figure 4c-d). We also studied FN, collagen-I, collagen-III, collagen-IV, MMP-2 and MMP-9 levels, which are markers for liver fibrosis. The results demonstrated a marked increase after treatment with PMA or LiCl (Figure 4e), which implied that captopril inhibited fibrosis through NF-κB and Wnt signaling pathway.

## Discussion

Our results demonstrate that captopril is a potentially effective anti-fibrosis reagent in HG-induced HSC-T6 cells and its effects are mediated by suppressing NF-kappaB and wnt3a/β-catenin. Additionally, captopril significantly increases cell viability and suppresses inflammation and oxidative stress. Use of NF-κB signaling pathway activator or Wnt signaling pathway activator confirms that NF-κB pathway and wnt3a/β-catenin pathway mediate the effects of captopril on oxidative stress, inflammation and fibrosis [23].

Liver fibrosis is a chronic process that usually progresses over decades and can eventually lead to cirrhosis [24]. Recently, more and more studies pay attention to the identification of drugs, which can produce inhibitory effects on the progression of hepatic fibrosis [25]. Captopril’s potent ant-fibrosis properties suggest that it is expected to be a potential anti-fibrosis agent. Captopril also suppresses oxidative stress and inflammation in HSC-T6 cells. Treatment of captopril significantly reduced MDA and ROS levels and increased SOD activities, as well as a decrease in inflammatory marker levels in HG-induced HSC-T6 cells. The mechanism of antioxidant activity and anti-inflammatory effects of captopril could be related to the inhibition of NF-kappaB and wnt3a/β-catenin. In previous research, captopril significantly increased Nrf2 and HO-1 protein levels, which could be involved in the modulation for oxidative stress in vitro [23]. We predicted that Nrf2 signaling pathway could be also engaged in mediating the effects of captopril on oxidative stress.

The roles of captopril in anti-oxidative stress and inflammation, as well as anti-fibrosis have been revealed in some studies [10,26,27]. Besides the regulatory role of NF-kappaB in inflammation and oxidative stress in diabetes, wnt3a/β-catenin was also reported to be involved in these processes [28–30]. Wnt3a activation in response to glucose was related to increased β-catenin level [31]. A study found that wnt3a/β-catenin pathway was engaged in hepatoprotective effects of hesperidin, which could be related to oxidative imbalance and inflammation [32]. Additionally, Wnt3a/β-catenin pathway was confirmed to modulate oxidative stress and inflammation [33–35]. Treatment with captopril contributes to the decrease in protein levels of FN, collagen I, collagen III, collagen IV, MMP-2 and MMP-9 in HG-induced HSC-T6 cells, which plays key roles in the formation of ECM through inducing the activation of HSCs [36–39]. In summary, captopril plays a protective role in protecting hepatic stellate cell from oxidative stress, inflammation and fibrogenesis injury caused by HG via NF-kappaB pathway and Wnt3a/β-catenin pathway.

## Conclusion

Liver injury is a typical inducement for the development of liver fibrosis and cirrhosis. Our study demonstrated that inhibition of the NF-kappaB pathway and Wnt3a/β-catenin by captopril under these HG conditions could exert inhibitory effects on fibrogenesis. The study provided experimental basis to confirm that captopril could be a potential therapeutic drug for the treatment of hepatic fibrosis. How captopril plays a role in vivo still requires amounts of experiments to explore, which will be considered in the next step of the study.

## Data Availability

The datasets used and/or analyzed during the current study are available from the corresponding author or first author on reasonable request.

## Highlights

1. Captopril reduces increased viability of rat hepatic stellate cells induced by HG stimulation.

2. Captopril alleviates oxidative stress, inflammation and fibrogenesis in HG-induced HSC-T6 cells.

3. These effects of captopril could be mediated by NF-κB pathway and wnt3α/β-catenin pathway.

## References

[1] Fujii H, Kawada N, Japan Study Group Of Nafld J-N. The Role of Insulin Resistance and Diabetes in Nonalcoholic Fatty Liver Disease. Int J Mol Sci. 2020;21(11):3863. 10.3390/ijms21113863 PMC7312931 32485838

[2] Zhao B, Li S, Guo Z, et al. Dopamine receptor D2 inhibition alleviates diabetic hepatic stellate cells fibrosis by regulating the TGF-β1/Smads and NFκB pathways. Clin Exp Pharmacol Physiol. 2021;48(3):370–380. 33179312 10.1111/1440-1681.13437

[3] Li Z, Li P, Lu Y, et al. A non-autonomous role of MKL1 in the activation of hepatic stellate cells. Biochim Biophys Acta Gene Regul Mech. 2019;1862(6):609–618. 30951901 10.1016/j.bbagrm.2019.03.001

[4] Sakai M, Sumiyoshi T, Aoyama T, et al. GPR91 antagonist and TGF-β inhibitor suppressed collagen production of high glucose and succinate induced HSC activation. Biochem Biophys Res Commun. 2020;530(2):362–366. 32798017 10.1016/j.bbrc.2020.07.141

[5] Wang Y, Sun Y, Zuo L, et al. ASIC1a promotes high glucose and PDGF-induced hepatic stellate cell activation by inducing autophagy through CaMKKβ/ERK signaling pathway. Toxicol Lett. 2019;300:1–9. 30291941 10.1016/j.toxlet.2018.10.003

[6] Mizar SMM, Kozman MR, Abo-Saif AA, et al. Combination of Captopril with Gliclazide Decreases Vascular and Renal Complications and Improves Glycemic Control in Rats with Streptozotocin-induced Diabetes Mellitus. Endocr Metab Immune Disord Drug Targets. 2021;21(6):1096-1106. 10.2174/1871530320666200821160436 32955003

[7] Ebadi Z, Moradi N, Kazemi Fard T, et al. Captopril and Spironolactone Can Attenuate Diabetic Nephropathy in Wistar Rats by Targeting microRNA-192 and microRNA-29a/b/c. DNA Cell Biol. 2019;38(10):1134–1142. 31433203 10.1089/dna.2019.4732

[8] Fouad AA, Al-Mulhim AS, Jresat I, et al. Protective effects of captopril in diabetic rats exposed to ischemia/reperfusion renal injury. J Pharm Pharmacol. 2013;65(2):243–252. 23278692 10.1111/j.2042-7158.2012.01585.x

[9] Gan Z, Huang D, Jiang J, et al. Captopril alleviates hypertension-induced renal damage, inflammation, and NF-κB activation. Braz J Med Biol Res. 2018;51(11):e7338. 30183974 10.1590/1414-431X20187338 PMC6125835

[10] Boskabadi J, Askari VR, Hosseini M, et al. Immunomodulatory properties of captopril, an ACE inhibitor, on LPS-induced lung inflammation and fibrosis as well as oxidative stress. Inflammopharmacology. 2019;27(3):639–647. 30291490 10.1007/s10787-018-0535-4

[11] Xie Y, Song T, Huo M, et al. Fasudil alleviates hepatic fibrosis in type 1 diabetic rats: involvement of the inflammation and RhoA/ROCK pathway. Eur Rev Med Pharmacol Sci. 2018;22:5665–5677. 30229844 10.26355/eurrev_201809_15834

[12] Zhang Y, Zhang L, Fan X, et al. Captopril attenuates TAC-induced heart failure via inhibiting Wnt3a/β-catenin and Jak2/Stat3 pathways. Biomed Pharmacother. 2019;113:108780. 30889487 10.1016/j.biopha.2019.108780

[13] Zhang C, Liu XQ, Sun HN, et al. Octreotide attenuates hepatic fibrosis and hepatic stellate cells proliferation and activation by inhibiting Wnt/β-catenin signaling pathway, c-Myc and cyclin D1. Int Immunopharmacol. 2018;63:183–190. 30098497 10.1016/j.intimp.2018.08.005

[14] Li W, Shen X, Wang Y, et al. The effect of Shengpuhuang-tang on retinal inflammation in streptozotocin-induced diabetic rats by NF-κB pathway. J Ethnopharmacol. 2020;247:112275. 31589966 10.1016/j.jep.2019.112275

[15] Zhang S, Xu L, Liang R, et al. Baicalin suppresses renal fibrosis through microRNA-124/TLR4/NF-κB axis in streptozotocin-induced diabetic nephropathy mice and high glucose-treated human proximal tubule epithelial cells. J Physiol Biochem. 2020;76(3):407–416. 32500512 10.1007/s13105-020-00747-z

[16] Wu Y, Wang Y, Liu B, et al. SN50 attenuates alveolar hypercoagulation and fibrinolysis inhibition in acute respiratory distress syndrome mice through inhibiting NF-κB p65 translocation. Respir Res. 2020;21(1):130. 32460750 10.1186/s12931-020-01372-6 PMC7251840

[17] Choi YJ, YH L, Lee ST. Galangin and kaempferol suppress phorbol-12-myristate-13-acetate-induced matrix metalloproteinase-9 expression in human fibrosarcoma HT-1080 cells. Mol Cells. 2015;38(2):151–155. 25518925 10.14348/molcells.2015.2229 PMC4332032

[18] Kong R, Kang OH, Seo YS, et al. MAPKs and NF‑κB pathway inhibitory effect of bisdemethoxycurcumin on phorbol‑12‑myristate‑13‑acetate and A23187‑induced inflammation in human mast cells. Mol Med Rep. 2018;17:630–635. 29115448 10.3892/mmr.2017.7852

[19] Song Y, Huang Q, Zhang Z, et al. Separate administration of ammonium pyrrolidinedithiocarbamate and phorbol myristate acetate at early and late stages decreases secondary brain injury following intracerebral haemorrhage in rats via the NF-κB pathway. Folia Neuropathol. 2020;58(2):166–175. 32729295 10.5114/fn.2020.96801

[20] Guo X, Chen Y, Hong T, et al. Induced pluripotent stem cell-derived conditional medium promotes Leydig cell anti-apoptosis and proliferation via autophagy and Wnt/β-catenin pathway. J Cell Mol Med. 2018;22(7):3614–3626. 29667777 10.1111/jcmm.13641 PMC6010900

[21] Li ZT, Zhang X, Wang DW, et al. Overexpressed lncRNA GATA6-AS1 Inhibits LNM and EMT via FZD4 through the Wnt/β-Catenin Signaling Pathway in GC. Mol Ther Nucleic Acids. 2020;19:827–840. 31981860 10.1016/j.omtn.2019.09.034 PMC6976905

[22] Zheng H, Jia L, Liu CC, et al. TREM2 Promotes Microglial Survival by Activating Wnt/β-Catenin Pathway. J Neurosci. 2017;37(7):1772–1784. 28077724 10.1523/JNEUROSCI.2459-16.2017 PMC5320608

[23] Bc T, Dj H, Wt L, et al. Functional potato bioactive peptide intensifies Nrf2-dependent antioxidant defense against renal damage in hypertensive rats. Food Res Int. 2020;129:108862. 32036911 10.1016/j.foodres.2019.108862

[24] Friedman SL. Mechanisms of hepatic fibrogenesis. Gastroenterology. 2008;134(6):1655–1669. 18471545 10.1053/j.gastro.2008.03.003 PMC2888539

[25] Schuppan D, Kim YO. Evolving therapies for liver fibrosis. J Clin Invest. 2013;123(5):1887–1901. 23635787 10.1172/JCI66028 PMC3635731

[26] Al-Hashem F, Al Humayed S, Haidara MA, et al. Captopril suppresses hepatic mammalian target of rapamycin cell signaling and biomarkers of inflammation and oxidative stress in thioacetamide-induced hepatotoxicity in rats. Arch Physiol Biochem. 2019;127(5):414-421. 10.1080/13813455.2019.1647249 31364422

[27] Kelleni MT, Sa I, Abdelrahman AM. Effect of captopril and telmisartan on methotrexate-induced hepatotoxicity in rats: impact of oxidative stress, inflammation and apoptosis. Toxicol Mech Methods. 2016;26(5):371–377. 27269004 10.1080/15376516.2016.1191576

[28] Bao L, Li J, Zha D, et al. Chlorogenic acid prevents diabetic nephropathy by inhibiting oxidative stress and inflammation through modulation of the Nrf2/HO-1 and NF-ĸB pathways. Int Immunopharmacol. 2018;54:245–253. 29161661 10.1016/j.intimp.2017.11.021

[29] Hu R, Wang MQ, Ni SH, et al. Salidroside ameliorates endothelial inflammation and oxidative stress by regulating the AMPK/NF-κB/NLRP3 signaling pathway in AGEs-induced HUVECs. Eur J Pharmacol. 2020;867:172797. 31747547 10.1016/j.ejphar.2019.172797

[30] Muriach M, Flores-Bellver M, FJ R, et al. Diabetes and the brain: oxidative stress, inflammation, and autophagy. Oxid Med Cell Longev. 2014;2014:102158. 25215171 10.1155/2014/102158 PMC4158559

[31] Chouhan S, Singh S, Athavale D, et al. Glucose induced activation of canonical Wnt signaling pathway in hepatocellular carcinoma is regulated by DKK4. Sci Rep. 2016;6(1):27558. 27272409 10.1038/srep27558 PMC4897783

[32] Zaghloul RA, Elsherbiny NM, Kenawy HI, et al. Hepatoprotective effect of hesperidin in hepatocellular carcinoma: involvement of Wnt signaling pathways. Life Sci. 2017;185:114–125. 28754618 10.1016/j.lfs.2017.07.026

[33] Wang Y, Wang Q, Li J, et al. Glutamine Improves Oxidative Stress through the Wnt3a/β-Catenin Signaling Pathway in Alzheimer’s Disease In Vitro and In Vivo. Biomed Res Int. 2019;2019:4690280. 31119171 10.1155/2019/4690280 PMC6500677

[34] Cui W, Zhang Z, Zhang P, et al. Nrf2 attenuates inflammatory response in COPD /emphysema: crosstalk with Wnt3a/β-catenin and AMP pathways. J Cell Mol Med. 2018;22(7):3514–3525. 29659176 10.1111/jcmm.13628 PMC6010849

[35] Liu Y, Wei M, Liu G, et al. Receptor-2 alleviates ox-LDL-induced lipid accumulation, inflammation and apoptosis via activation of Wnt/β-catenin signaling. Gen Physiol Biophys. 2020;39(5):437–448. 33084597 10.4149/gpb_2020014

[36] Mu M, Zuo S, Wu RM, et al. Ferulic acid attenuates liver fibrosis and hepatic stellate cell activation via inhibition of TGF-β/Smad signaling pathway. Drug Des Devel Ther. 2018;12:4107–4115. 10.2147/DDDT.S186726 PMC6284527 30584275

[37] Munsterman ID, Kendall TJ, Khelil N, et al. Extracellular matrix components indicate remodelling activity in different fibrosis stages of human non-alcoholic fatty liver disease. Histopathology. 2018;73(4):612–621. 29856896 10.1111/his.13665

[38] Yu DK, Zhang CX, Zhao SS, et al. The anti-fibrotic effects of epigallocatechin-3-gallate in bile duct-ligated cholestatic rats and human hepatic stellate LX-2 cells are mediated by the PI3K/Akt/Smad pathway. Acta Pharmacol Sin. 2015;36(4):473–482. 25832428 10.1038/aps.2014.155 PMC4387300

[39] Perumal N, Perumal M, Halagowder D, et al. Morin attenuates diethylnitrosamine-induced rat liver fibrosis and hepatic stellate cell activation by co-ordinated regulation of Hippo/Yap and TGF-β1/Smad signaling. Biochimie. 2017;140:10–19. 28552397 10.1016/j.biochi.2017.05.017

